# Supplementary material for: Ultrafast coherence transfer in DNA-templated silver nanoclusters
Source: Nat Commun. 2017 May 26;8:15577. doi: 10.1038/ncomms15577 (PMC5493596; doi:10.1038/ncomms15577)
Supplement: Supplementary Information — Supplementary Figures, Supplementary Notes and Supplementary References [file ncomms15577-s1.pdf]

Supplementary Note 1. Polarization Spectroscopy

2D Anisotropy

We define the 2D anisotropy entirely in analogy to the pump-probe- and fluorescence-anisotropy as:

$$\boldsymbol{r}_0 = \frac{\langle 0,0,0,0\rangle - \langle \frac{\pi}{2}, \frac{\pi}{2}, 0,0\rangle}{\langle 0,0,0,0\rangle + 2\langle \frac{\pi}{2}, \frac{\pi}{2}, 0,0\rangle} = \frac{LD}{3MA}$$

Where $\langle \theta_1, \theta_2, \theta_3, \theta_4 \rangle$ represents the four-pulse (including LO) sequence with polarization angles θ_i for the ith pulse (note that a linear polarizer set to θ_4 is placed in the path of the emitted signal as well), LD is the linear dichroism, and MA is the spectrum recorded at the magic angle condition $\langle 54.7°, 54.7°, 0,0\rangle$.

- It may be difficult to distinguish low-anisotropy features (*i.e.* cross-peaks between states with relatively large projection angles) from features resulting from several overlapping transitions.
- Due to the division by the MA spectrum, the anisotropy diverges in regions with no absorbance and in regions where ESA and GSB overlap so as to result in a net ΔOD of 0.
- In the presence of coherences between non-parallel states (typically at very early times) the relationship between the experimentally determined anisotropy and the projection angles may be a relatively complex function, which has to be analysed on a case-to-case basis.[1, 2]

We thus use the anisotropy map for qualitative analysis of the system, and analyse the spectral structure only after the decay of any possible electronic coherence. We further mask off the anisotropy amplitude scale in regions with low (or no) absorbance, so as to avoid diverging values.

Constructing the cross-peak specific map

The amplitude of a feature in the 2D map is a function of not only the transition moment strengths of the involved transitions, but also their relative angles. In the following analysis we consider the map as a grid of "two-colour pump-probe" experiments – each spectral point is the result of "pumping" one transition at some ω_1 frequency, and "probing" another (or the same in the case of the diagonal features) along some ω_3 frequency. The general expression for the signal amplitude resulting from a sequence of four polarized pulses $\mathbf{e_n}$ interacting with four transition moments $\mathbf{q_n}$ in isotropic solution has been derived by Hochstrasser and coworkers[3, 4, 5].

eq 1. $$S = \overline{\begin{bmatrix} (\boldsymbol{q_1 q_2})(\boldsymbol{q_3 q_4}) \\ (\boldsymbol{q_1 q_3})(\boldsymbol{q_2 q_4}) \\ (\boldsymbol{q_1 q_4})(\boldsymbol{q_2 q_3}) \end{bmatrix}} \begin{bmatrix} 4 & -1 & -1 \\ -1 & 4 & -1 \\ -1 & -1 & 4 \end{bmatrix} \begin{bmatrix} (\boldsymbol{e_1 e_2})(\boldsymbol{e_3 e_4}) \\ (\boldsymbol{e_1 e_3})(\boldsymbol{e_2 e_4}) \\ (\boldsymbol{e_1 e_4})(\boldsymbol{e_2 e_3}) \end{bmatrix}$$

We consider only features resulting from two (or one) distinct transition moments. Further, we are interested in static and energy-transfer signals rather than coherent signals, and thus consider signals resulting from interactions where $\mathbf{q_1} = \mathbf{q_2}$ and $\mathbf{q_3} = \mathbf{q_4}$.

The transition $\mathbf{q_1}$ can be decomposed into two spectral projections: $\mathbf{q_z}$ parallel to the "probe" $\mathbf{q_3}$, and $\mathbf{q_x}$ perpendicular to $\mathbf{q_3}$. The signal amplitude in Eq. 1 then appears as:

eq 2. $$S = \left(q_x^2 q_3^2 \overline{\begin{bmatrix} 1 \\ 0 \\ 0 \end{bmatrix}} + q_z^2 q_3^2 \overline{\begin{bmatrix} 1 \\ 1 \\ 1 \end{bmatrix}} \right) \begin{bmatrix} 4 & -1 & -1 \\ -1 & 4 & -1 \\ -1 & -1 & 4 \end{bmatrix} \begin{bmatrix} (\boldsymbol{e_1 e_2})(\boldsymbol{e_3 e_4}) \\ (\boldsymbol{e_1 e_3})(\boldsymbol{e_2 e_4}) \\ (\boldsymbol{e_1 e_4})(\boldsymbol{e_2 e_3}) \end{bmatrix}$$

Using the pulse polarization sequences $VV = \langle 0,0,0,0 \rangle$ and $VH = \langle \frac{\pi}{2}, \frac{\pi}{2}, 0,0 \rangle$ yields the signal amplitudes:

eq 3. $$S_{VV} = 2q_3^2(q_x^2 + 3q_z^2) \; ; \; S_{VH} = 2q_3^2(2q_x^2 + q_z^2)$$

These signal amplitudes can be used to generate the 2D analogue to the pump-probe (or fluorescence) anisotropy in terms of the spectral projections

eq 4. $$r_0 = \frac{S_{VV}-S_{VH}}{S_{VV}+2S_{VH}} = \frac{2q_z^2-q_x^2}{5(q_z^2+q_x^2)}$$

Applying the normalization condition $q_x^2 + q_z^2 = 1$ and reorganizing allows the construction of expressions for these squared dipole moment projections (i.e. oscillator strengths) in terms of the experimentally measurable anisotropy:

eq 5. $$q_x^2 = A_{par} = \frac{1}{3}(2-5r_0)\ ;\ q_z^2 = A_{perp} = \frac{1}{3}(5r_0+1)$$

These expressions can be multiplied by the magic angle spectrum to generate the full 2D spectra containing only the projections into the "probe" direction (i.e. parallel to the diagonal), and the projection perpendicular to the diagonal. This latter projection, shown in the manuscript Figure 2B, in particular is of interest as it entirely eliminates the diagonal features from the spectrum, allowing clearer view of many cross-peaks due to significant reduction in spectral congestion. It should be noted that this projection is formally equivalent to the "cross-peak specific" spectrum S_{VV}-3S_{VH} used by Fleming and coworkers[6, 7].

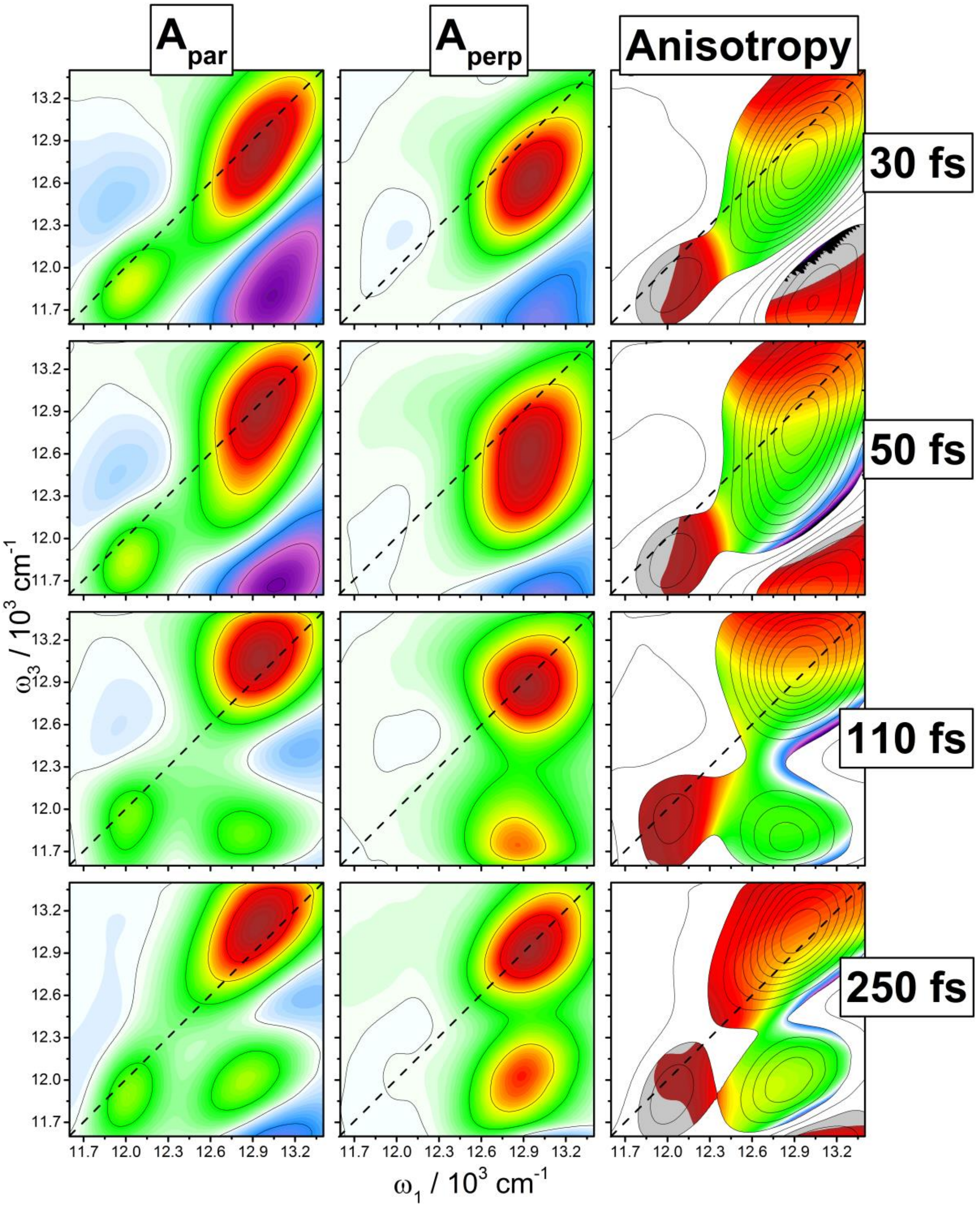

Supplementary Figure 1: Spectral projections A_{par} and A_{perp} (see eq. 5) and 2D anisotropy at early population times. A coherence contribution with 800 fs dephasing time has been taken into account in calculating the anisotropy. The colour scales are defined in Figure 2 in the main text.

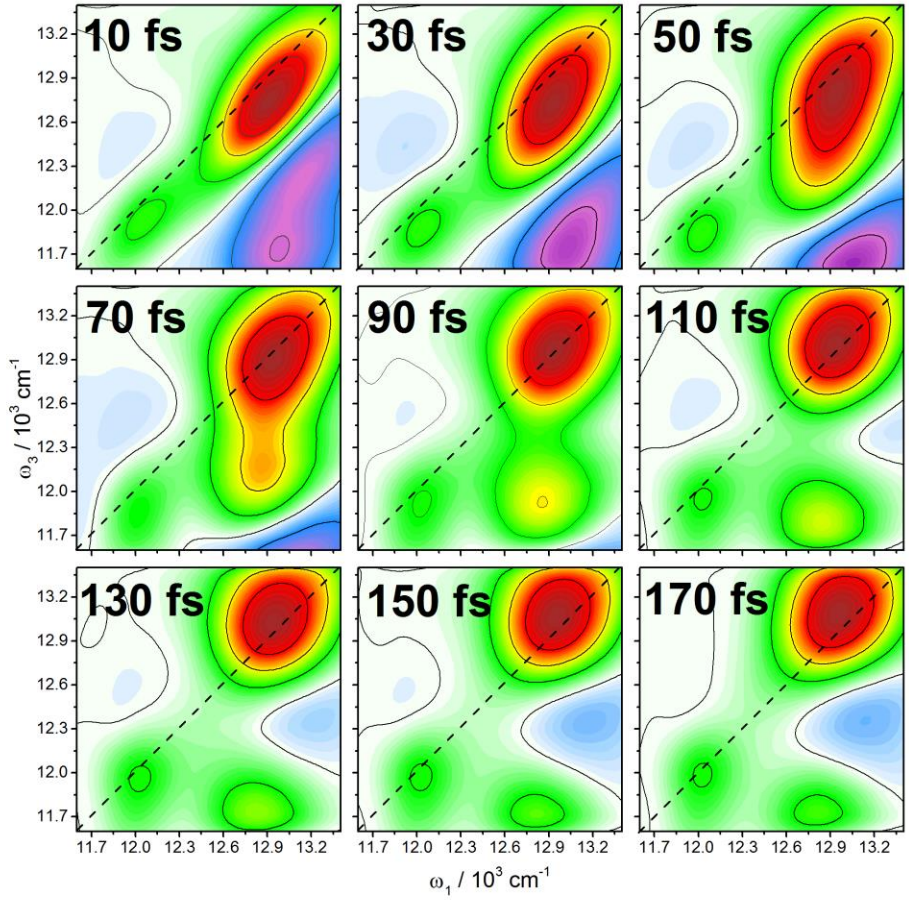

Supplementary Figure 2: Magic angle polarization condition (total real part) 2D spectra at selected short times shows the initial steps in the H-to-L band relaxation process.

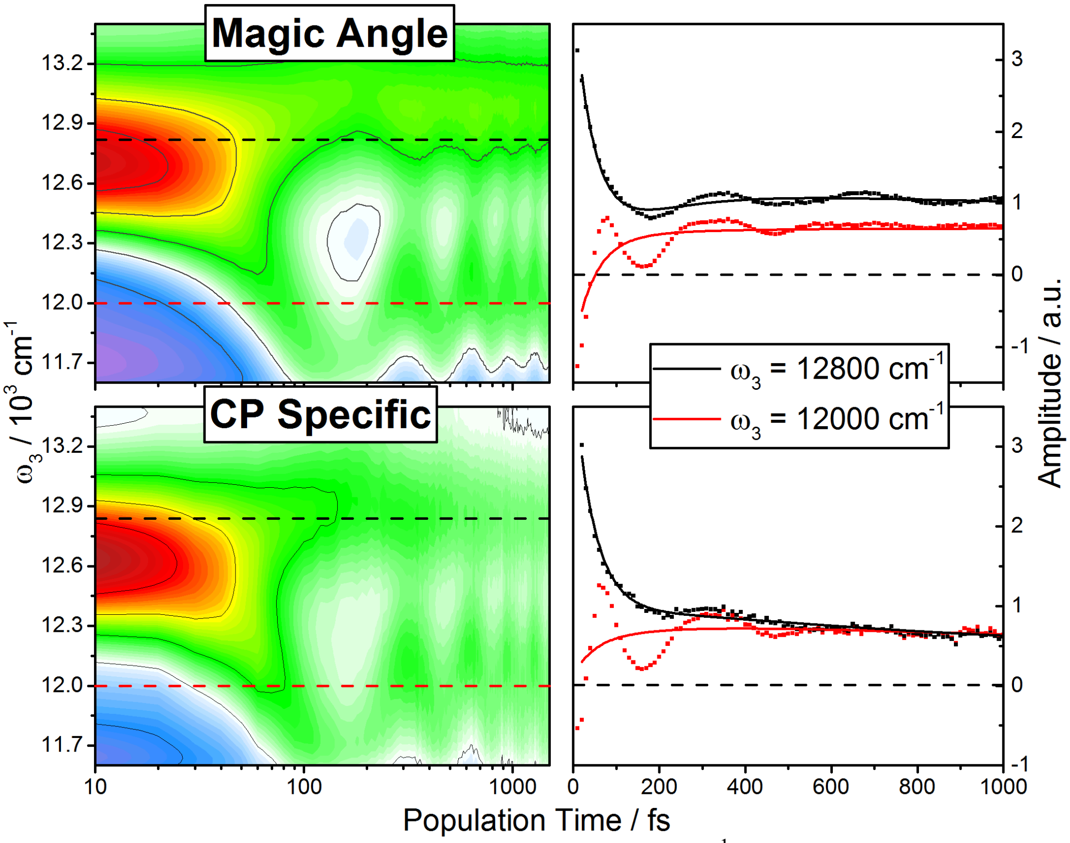

Supplementary Figure 3: ω_3 slices extracted at $\omega_1 = 12860$ cm^{-1} shown as a function of population time (left). Single-point kinetics at the diagonal ($\omega_3 = 12860$ cm^{-1}, black) and L-band equilibrium position ($\omega_3 = 12000$ cm^{-1}, red) show complex, multi-exponential dynamics (and oscillations). The dominating short-time component is ~50 fs, and can be assigned to the H-to-L population transfer.

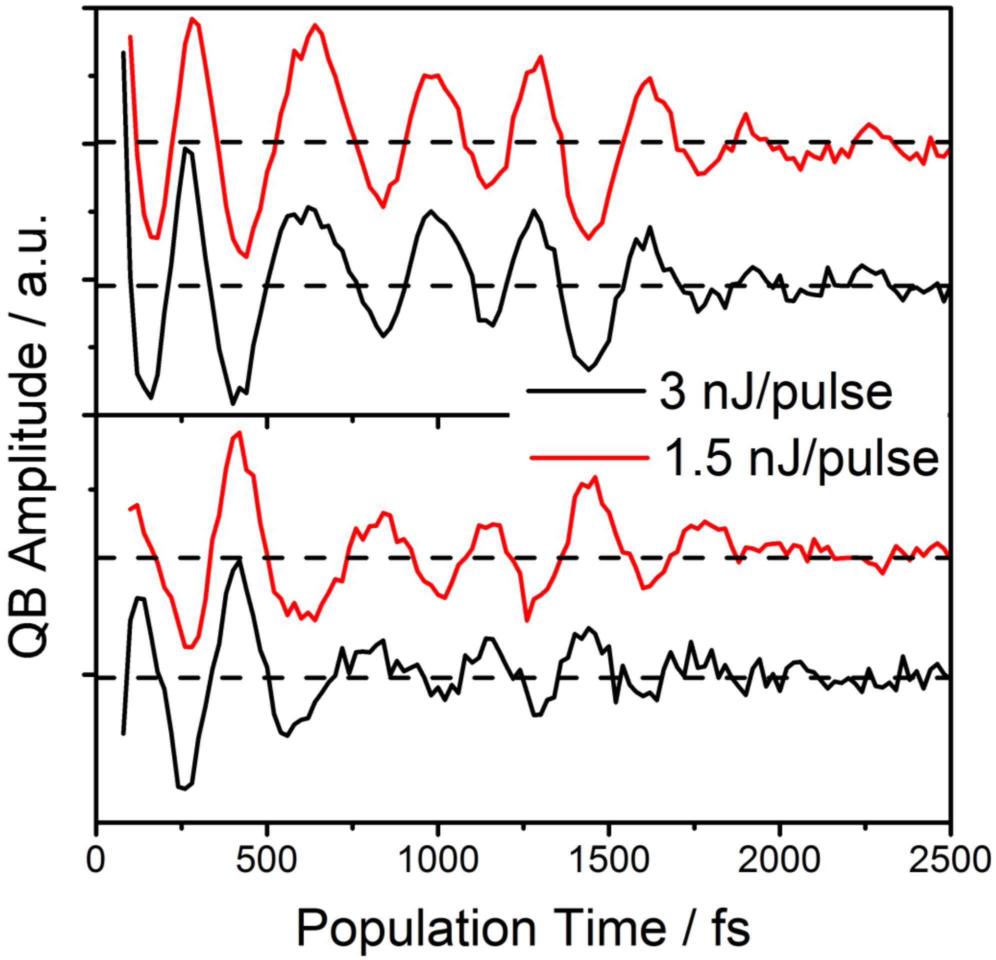

Supplementary Figure 4: Pulse-energy dependence of the quantum beats. The traces shown are residuals of the rephasing signal kinetics above and below the H-L cross-peak, shown at the top and bottom of the figure, respectively, after subtraction of exponential dynamics. The QB frequency shows no pulse energy dependence in this range.

Supplementary Note 2. Quantum Beat Analysis

Positive/Negative Frequency Quantum Beats in a Displaced Oscillator[8]

Quantum beats (QBs) as observed in pump-probe and 2D spectra result from superpositions of quantum mechanical states – *e.g.* states **a** (lower energy) and **b** (higher energy). These superpositions are created by the interaction of the excitation field(s) with the transition dipole moment of these states. QBs in the signal will appear at the frequency difference $|\omega_{ab}|$ between the states. Depending on the order of interactions, the sequence: interaction first with **a** and then with **b** will result in a QB time evolution $\propto e^{+i\omega_{ab}t}$, while the sequence **b** first and then **a** results in a time evolution $\propto e^{-i\omega_{ab}t}$. Both terms will contribute, potentially resulting in interference. We list double-sided Feynman diagrams[9] for all GSB and SE coherence pathways for a simple displaced oscillator in Supplementary Figure 5. Each diagram is assigned a sign according to whether it corresponds to a QB with population time evolution proportional to $e^{+i\omega_{ab}t}$ or $e^{-i\omega_{ab}t}$. We schematically show *where* these pathways contribute in the 2D spectrum in Supplementary Figure 6. In all diagrams subscript indexes correspond to vibrational excitations.

GSB pathways appear only with a single frequency sign: negative in rephasing and positive in non-rephasing. The SE pathways on the other hand appear with both signs and pairwise identical amplitudes. This can be used to discriminate ground- and excited-state coherences. Note that the "pattern" of QB amplitudes and signs are identical for pure GSB and SE contributions if the spectrum is not separated into rephasing and non-rephasing parts, making pure excited-state and pure ground-state coherence indistinguishable in this simple model. In real systems, effects such as significant Stokes shifts and shifts in vibrational frequency may allow separation of these contributions.

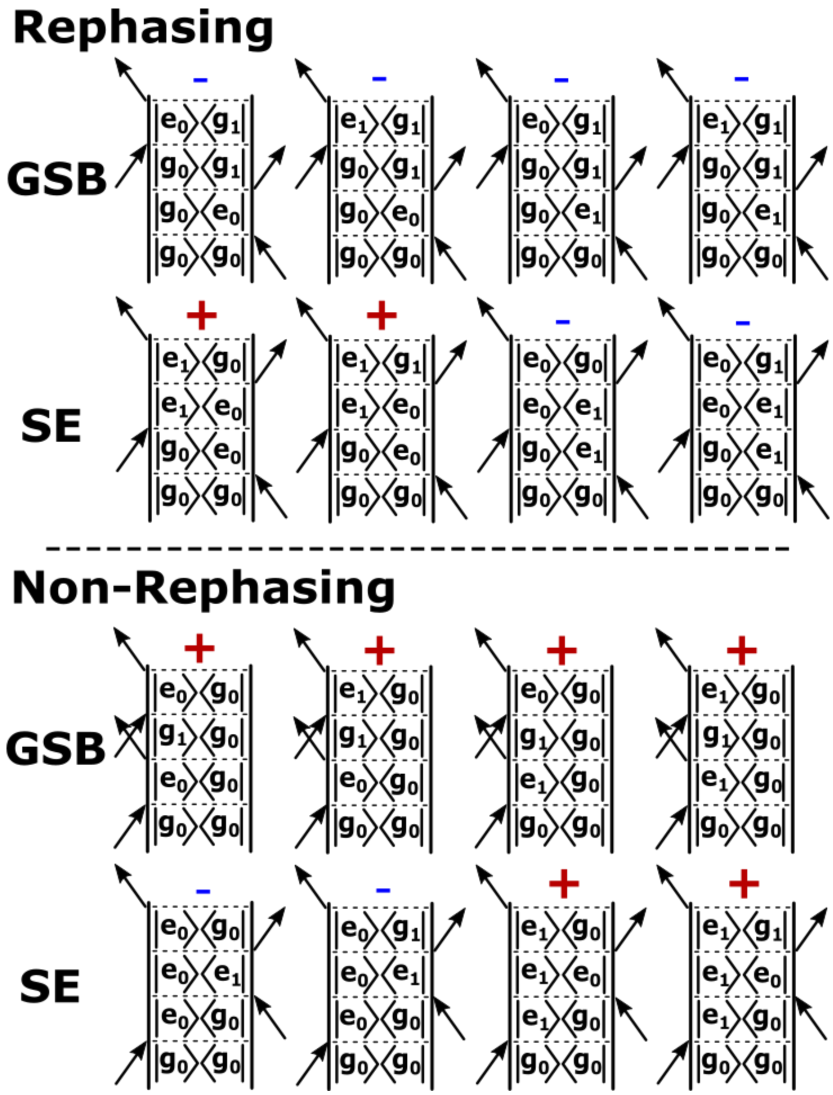

Supplementary Figure 5: Rephasing and non-rephasing coherent pathways in the displaced oscillator, labelled with frequency signs (see text). ESA pathways not shown.

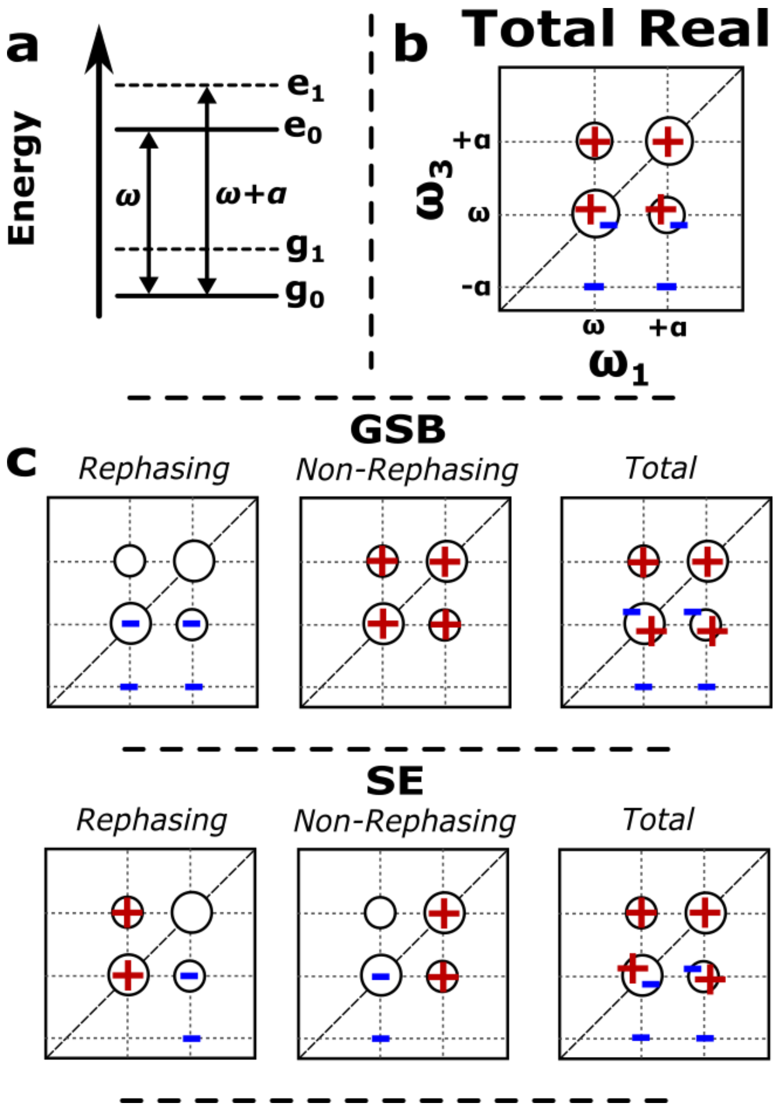

Supplementary Figure 6: **a**: Two-state displaced oscillator model. **b**: Schematic QB amplitude map showing where beats will appear, and with which frequency sign, after Fourier transforming total real data. **c**: The total QB response separated into individual GSB and SE components. The QB amplitude "patterns" for GSB and SE contributions are identical in total real data.

Three-state system

The $Ag_{20}NC$ electronic structure involve (at least) three excited electronic levels: **L**, and the two **H** levels, schematically shown in Supplementary Figure 7. A number of coherent pathways analogous to the ones observed in the displaced two-state oscillator may be expected. For low vibrational frequencies these will contribute around the diagonal features, essentially giving the response of two displaced oscillators at different energies. In addition to these "trivial" coherent pathways, coherences can be induced in one state and probed in the other. In particular, ground state vibrational coherences induced by excitation into e.g. **H**could possibly be observable around **L** in the case of a shared ground-state. Importantly, these pathways will still appear with the ω_2 frequency sign of a ground-state coherence (negative in rephasing, positive in non-rephasing). The experimental observation is however equal amplitude quantum beats in positive and negative frequencies; induced by excitation in **H** and probed around **L**. This is clear evidence for excited state coherence. In Supplementary Figure 7 we show the Feynman diagrams involving the creation of coherence in the **H** states, followed by rapid (relative to the period) transfer to vibrational coherence in **L**. In Supplementary Figures 8 and 9 we schematically show the spectral positions where these pathways are expected to contribute, and compare this to the experimental data. Overall we find excellent agreement, lending strong support to the idea of ultrafast coherence transfer in $Ag_{20}NC$.

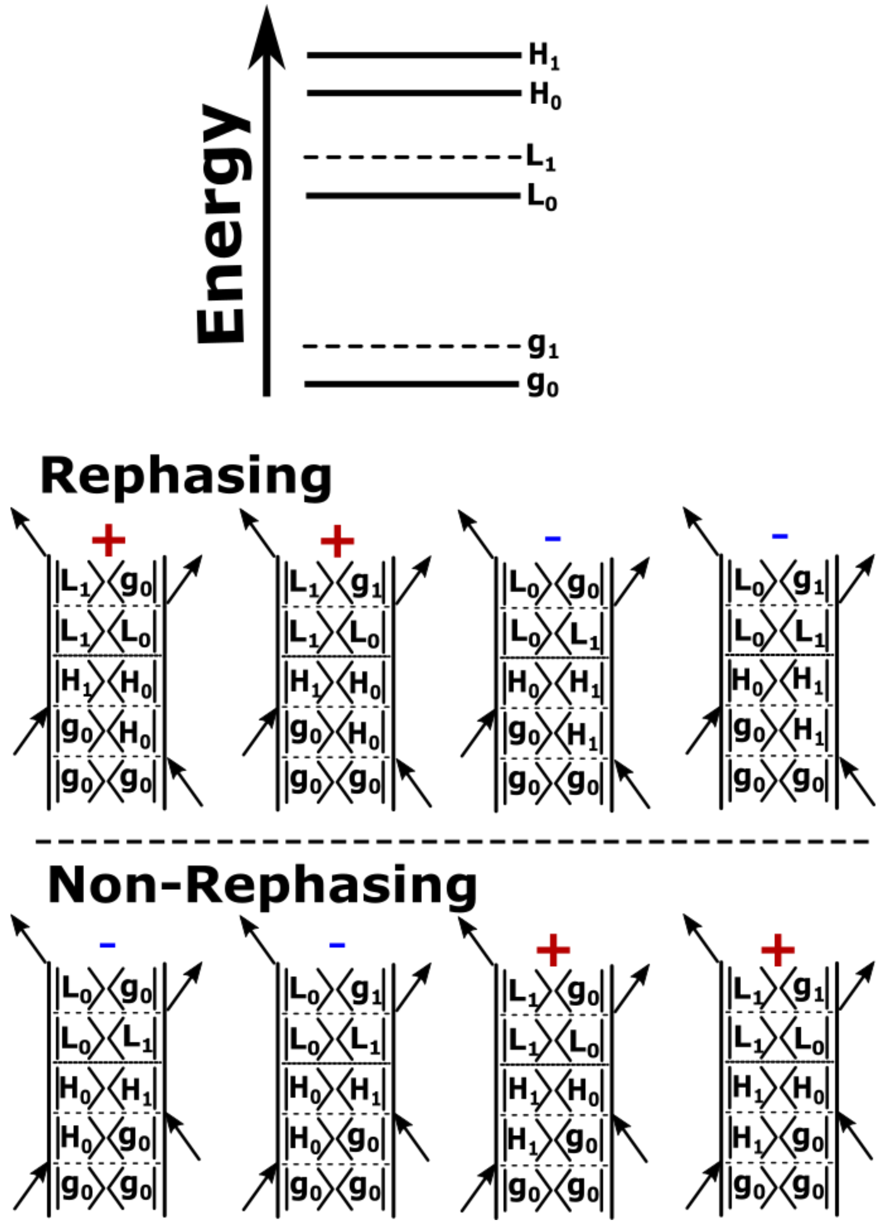

Supplementary Figure 7: Top: Qualitative energy-level structure representation for Ag_{20}NC. Bottom: SE pathways involving transfer of coherence from the H band to L.

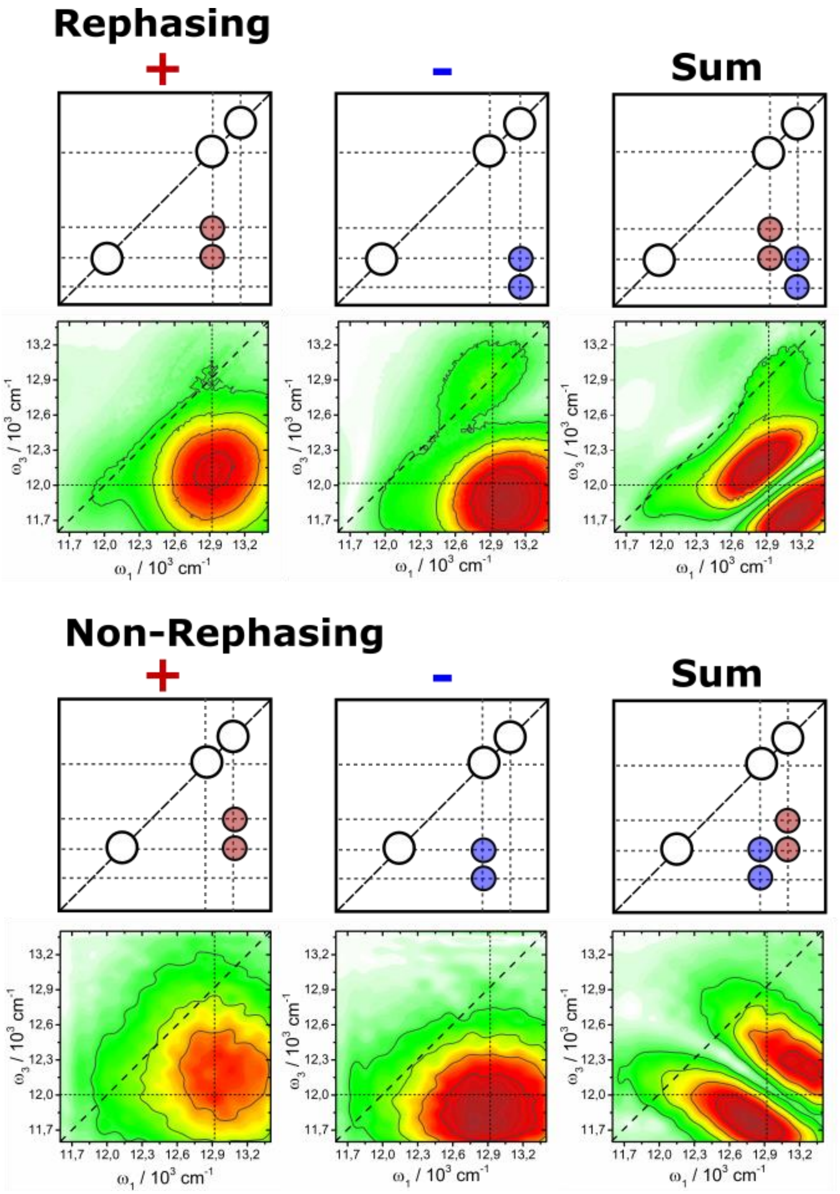

Supplementary Figure 8: Schematic representation of the spectral position and frequency sign of the stimulated emission coherence transfer pathways in comparison with positive and negative frequency components of the complex Fourier transformed data. Rightmost (Sum) column is the maps resulting from a real Fourier transform of the data.

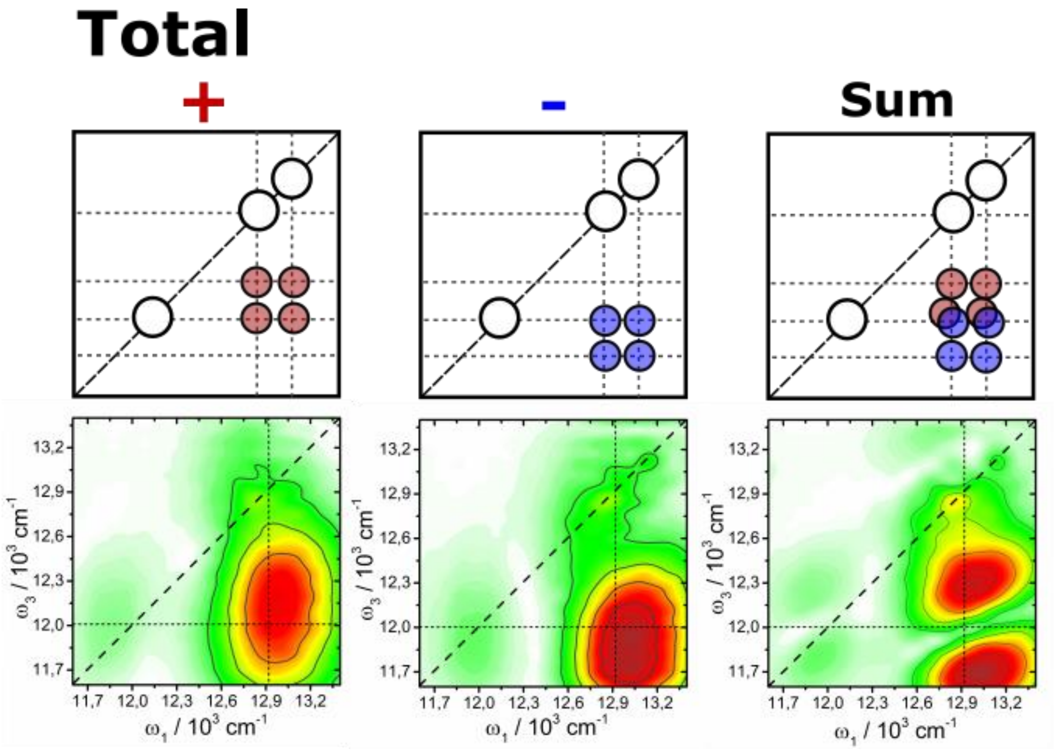

Supplementary Figure 9: Schematic representation of positive and negative frequency stimulated emission quantum beat contributions compared with experimental data. Positive and negative frequency components are extracted from a complex Fourier transform of the data. The sum spectrum is equivalent to the spectrum resulting from a Fourier transform of absorptive data, and is (in this model) indistinguishable from a pure ground-state vibration.

Supplementary Note 3. Synthesis and Characterization

DNA-AgNC synthesis

Single stranded DNA (IDT, standard desalting) with a sequence CCCACCCACCCTCCCA was diluted in 0.1 M citrate buffer (pH 6.2) to give [DNA] = 6.67 mM. The diluted DNA was heated to 80-85 °C in order to start with a homogeneous dilution of single stranded DNA.[10] The solution was then cooled to room temperature. Silver nitrate (99.9999 %, Sigma Aldrich) was diluted in Milli-Q water (MQ) to a concentration of [$AgNO_3$] =26.7 mM and added to the DNA solution in a molar ratio of DNA: $AgNO_3$ of 1:8. The sample was then reduced by adding a fresh solution of $NaBH_4$ (99.99 %, Sigma Aldrich) in MQ with a concentration of [$NaBH_4$] = 16.4 mM and a final molar ratio of DNA: $AgNO_3$: $NaBH_4$ of 1:8:4.

DNA-AgNC purification

The synthesized IR silver nanoclusters were purified by HPLC purification to remove all remaining DNA and unwanted silver nanoclusters. An analytical HPLC Dionex UltiMate 3000 system with a Dionex UltiMate 3000 fluorescence detector and Phenomenex Germini C18 column (5μm, 110Å, 50 × 4.6 mm) was used for purification. The mobile phase in the HPLC purification consisted of 35mM triethylammonium acetate (TEAA) in water (solvent A) and methanol (solvent B) at pH 7. A linear solvent gradient was used with an increase of solvent B from 5-50% over 10 minutes followed by 5 minutes wash at 95% methanol. After purification the samples were concentrated ~5x by centrifugal filtration using Pur-A-Lyzer maxi 1200 dialysis kit with a capacity of 0.1-3 mL and a molecular weight cut-off at 12-14 kDa (SigmaAldrich).

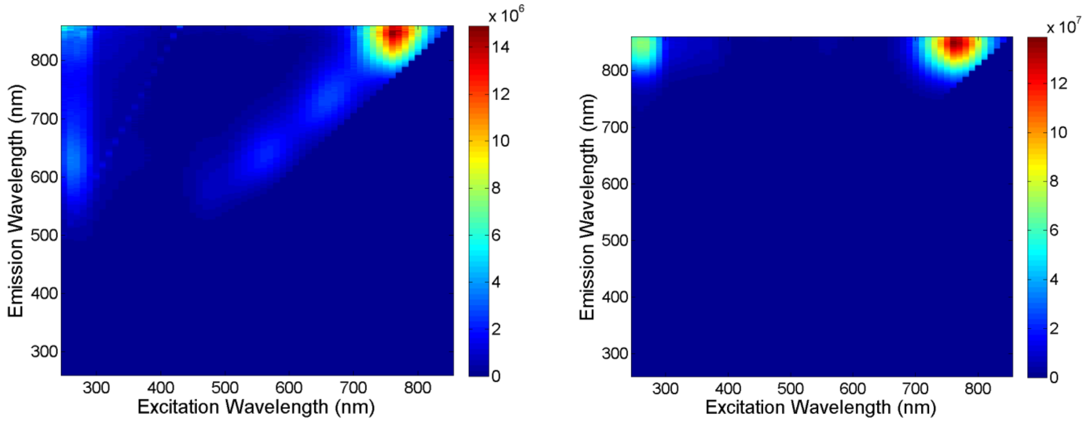

Supplementary Figure 10: Excitation-emission 2D scans before (left) and after (right) HPLC purification. The colour bar indicates the fluorescence intensity in arbitrary units. The data was recorded using a QuantaMaster^{TM}400 (PTI) fluorometer.

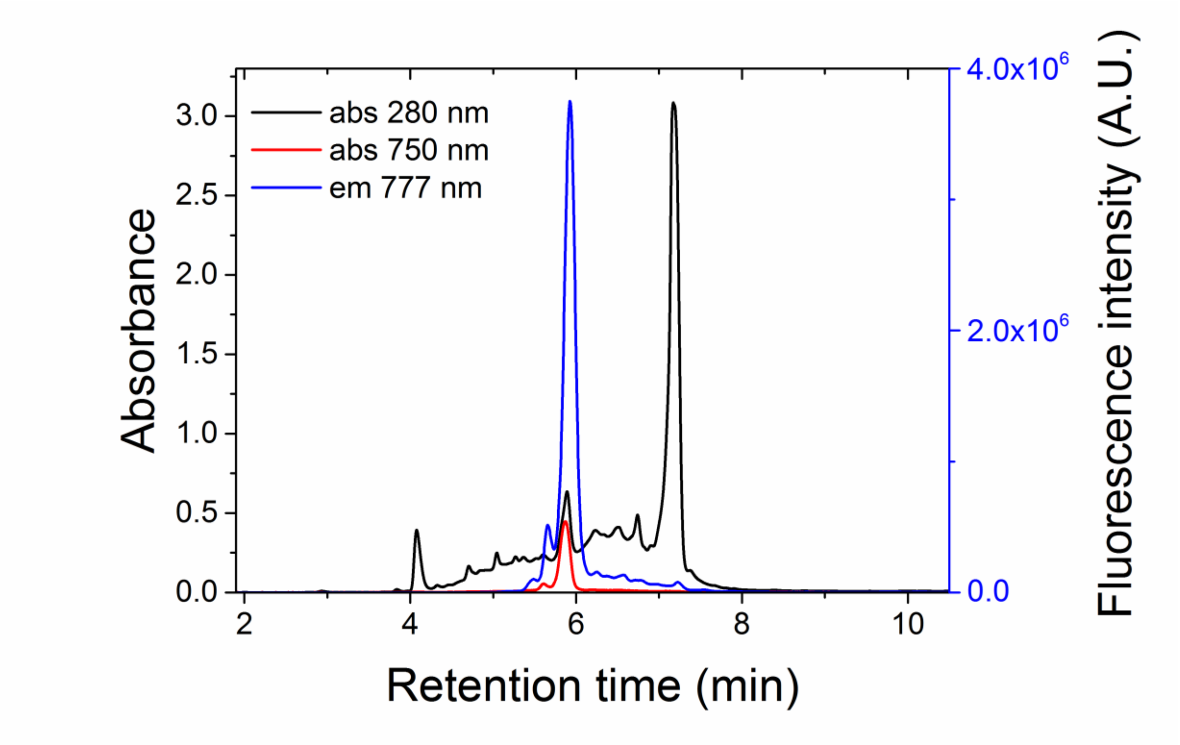

Supplementary Figure 11: HPLC chromatogram showing the retention time of DNA monitored by the absorbance at 280 nm and the retention time of the near IR emitting silver nanoclusters, monitored by the absorbance at 750 nm and the fluorescence at 777 nm (upon excitation at 270 nm).

Supplementary References

1. Savikhin S, Buck DR, Struve WS. Oscillating anisotropies in a bacteriochlorophyll protein: Evidence for quantum beating between exciton levels. *Chem. Phys.* **223**, 303-312 (1997).

2. Smith ER, Jonas DM. Alignment, Vibronic Level Splitting, and Coherent Coupling Effects on the Pump-Probe Polarization Anisotropy. *J. Phys. Chem. A* **115**, 4101-4113 (2011).

3. Hochstrasser RM. Two-dimensional IR-spectroscopy: polarization anisotropy effects. *Chem. Phys.* **266**, 273-284 (2001).

4. Zanni MT, Ge NH, Kim YS, Hochstrasser RM. Two-dimensional IR spectroscopy can be designed to eliminate the diagonal peaks and expose only the crosspeaks needed for structure determination. *Proc. Natl. Acad. Sci. USA* **98**, 11265-11270 (2001).

5. Hamm P, Zanni MT. *Concepts and methods of 2d infrared spectroscopy*. Cambridge University Pres (2011).

6. Read EL*, et al.* Cross-peak-specific two-dimensional electronic spectroscopy. *Proc. Natl. Acad. Sci. USA* **104**, 14203-14208 (2007).

7. Read EL, Schlau-Cohen GS, Engel GS, Wen JZ, Blankenship RE, Fleming GR. Visualization of excitonic structure in the Fenna-Matthews-Olson photosynthetic complex by polarization-dependent two-dimensional electronic spectroscopy. *Biophys. J.* **95**, 847-856 (2008).

8. Li HB, Bristow AD, Siemens ME, Moody G, Cundiff ST. Unraveling quantum pathways using optical 3D Fourier-transform spectroscopy. *Nat. Commun.* **4**, (2013).

9. Mukamel S. *Principles of nonlinear optical spectroscopy*. Oxford University Press (1995).

10. Kibbe WA. OligoCalc: an online oligonucleotide properties calculator. *Nucleic Acids Res.* **35**, W43-46 (2007).
